# Climate change influences on crop mix shifts in the United States

**DOI:** 10.1038/srep40845

**Published:** 2017-01-18

**Authors:** Sung Ju Cho, Bruce A. McCarl

**Affiliations:** 1 Korea Rural Economic Institute, 601 Bitgaram-ro, Naju-si, Jeollanam-do, 58217, Korea; 2 Department of Agricultural Economics, Texas A&M University, College Station, TX 77843-2124, USA

## Abstract

We examine the impact of current and future climate on crop mixes over space in the US. We find using historical data that temperature and precipitation are among the causal factors for shits in crop production location and mixes, with some crops being more sensitive than others. In particular, we find that when temperature rises, cotton, rice, sorghum and winter wheat are more likely to be chosen. We also find that barley, sorghum, winter wheat, spring wheat and hay are more likely to be chosen as regions become drier, and corn, cotton, rice and soybeans are more likely to be selected in wetter regions. Additionally, we assess how much of the observed crop mix shifts between 1970 and 2010 were contributed to by climate change. There we find climate explains about 7–50% of the shift in latitude, 20–36% in longitude and 4–28% of that in elevation. Finally, we estimate climate change impacts on future crop mix under CMIP5 scenarios. There we find shifts in US production regions for almost all major crops with the movement north and east. The estimates describe how the farmers respond to altering climate and can be used for planning future crop allocations.

Crop mixes, changing land allocations for various crops, are an often discussed climate change adaptation strategy^1^,^2^,^3^,^4^,^5^. Attavanich *et al*.^2^ and Reilly *et al*.^3^ examined historical shifts in major US crops arguing that climate change is a causal factor. Examining crop mixes gives a sense of adaptation as land use shifts away from more vulnerable crops. In fact looking at individual crops one at a time in climate change analysis can be misleading. While simulation studies like Rosenzweig and Parry^6^ or econometric studies like Schlenker and Roberts^7^ show concern about crop yield vulnerability, they overstate agricultural vulnerability since producers can adapt by shifting crop mix. Although land value studies like those by Mendelsohn *et al*.^8^ implicitly treat adaptations including crop mix shifts, they do not address what crops may shift. Foreseeing such shifts is a factor in planning of future transportation, processing capacity and other infrastructure (see the findings in Attavanich *et al*.^2^).

This study examines how land use shares of US crops have been affected by past climate change and then projects shifts into the future. The analysis is done at the county level employing the results of estimated crop mix share equations.

## Estimation procedures

To estimate climate effects on crop mix, we estimate land use shares by crop using maximum quasi-likelihood estimation for multinomial fractional regression following Koch^9^, Kala *et al*.^10^, and Murteira and Ramalho^11^. Details on the exact method used are in the supplemental information.

The estimation covers planted acreage of barley, corn, upland cotton, rice, sorghum, soybeans, winter wheat, spring wheat, and alfalfa hay, which jointly occupy of about 96% of US cropland. The data used cover these crops in 2693 counties in 41 US states over the years 1975 to 2011. The data used in the estimation were drawn from USDA NASS Quick Stats^12^. The total number of observations is 99,641.

The independent variables not only involve climate characteristics but also revenue by crop, land capability, human population density, total planted acres, average elevation and proportion of irrigated land. The climate attributes involve annual average temperature and annual total precipitation in linear and squared form plus monthly standard deviations of temperature and precipitation. Additionally, the Palmer drought severity index (PDSI) was used with negative values indicating dry conditions and positive values indicating wet ones. Most of the independent variables were formed as lagged 5-year averages in an attempt to model farmer reactions to longer term climate and other effects. Time-invariant factors include land capability and average elevation of lands for each county.

## Results

### Estimation results

The average partial effects of the independent variables computed from the estimated model appear in Table 1. The results show: (1) The acreage shares of all major crops are affected by changes in temperature and precipitation. Upland cotton, rice, sorghum and winter wheat are more likely to be chosen when the 5-year average temperature increases. On the other hand, barley, corn, soybeans, spring wheat and alfalfa hay are less likely to be chosen when the temperature increases. When annual precipitation increases, land use shares for corn, upland cotton, rice and soybeans increase while shares for barley, sorghum, hay and both types of wheat decline; (2) Increases in the annual standard deviation of temperature reduces the share of barley, rice, winter wheat and hay, while larger standard deviations for precipitation decrease the shares for corn, cotton and hay. (3) The crop mix shares positively respond to increases in own crop net returns. (4) Mixed effects occur when human population density increases, with barley, corn and soybeans increasing. (5) As total planted area increases, cotton, rice, soybeans, winter wheat and spring wheat shares grow. For instance, in Corn Belt regions with relatively cold temperature, fluent precipitation and high irritation rate, corn is likely to be more chosen than other crops although the non-linear relationships between variables indicate that too high or low values of climate variables have limitations to the degrees of changes.

**Figure 1:**
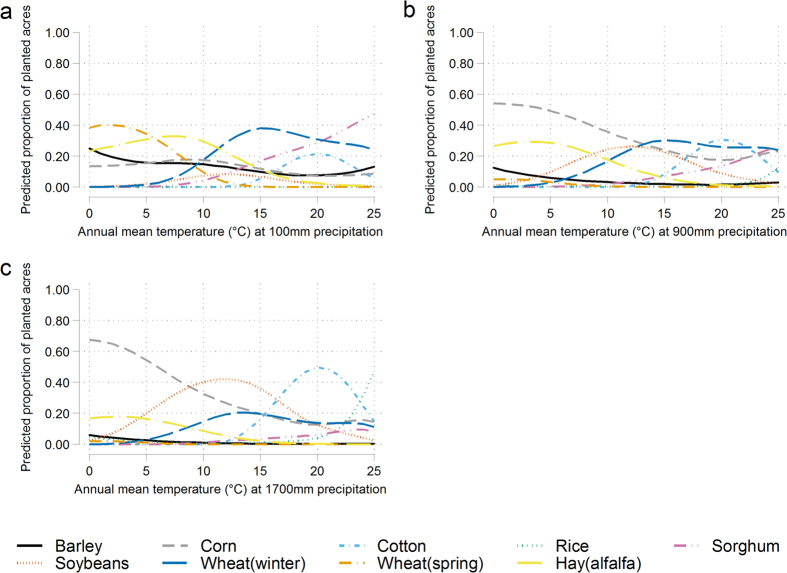
Response of predicted proportions of crop planted acres to temperature changes under different precipitations. (**a**) Given 100 mm annual precipitation, the most selected crops are spring wheat and hay in lower temperature, winter wheat in moderate temperature, and sorghum in higher temperature. (**b**) Given 900 mm annual precipitation, the most selected crops are corn in lower temperature, winter wheat, corn and soybeans in moderate temperature, and upland cotton in higher temperature. (**c**) Given 1700 mm annual precipitation, the most selected crops are corn in lower temperature, soybeans in moderate temperature, and upland cotton and rice in higher temperature.

Figure 1 shows how the predicted crop shares vary with temperature for three different precipitation cases. There we see (1) Spring wheat and hay are most likely to be chosen in places with low precipitation, with corn most likely under moderate or high precipitation. (2) In higher temperature instances, sorghum is favored under low precipitation, but cotton is more likely to be planted under moderate and high precipitation. (3) As precipitation goes up, the most selected crop in moderate temperature change from winter wheat to soybeans. Hay, winter wheat, spring wheat and sorghum are more likely to be chosen in drier regions, and corn, soybeans and cotton are more likely to be selected in wetter regions.

### Climate contribution to historical shifts

Now we examine how much climate change has contributed to the way US crop mixes have shifted since 1970. We do this using a production weighted average centroid approach as in Reilly *et al*.^3^ although we also include elevation.

Table 2 presents ways that the crop mix shares have changed since 1970 and the amount of this change that arises due to climate change as evaluated through the estimated equation. Overall, climate is substantially responsible for the westward movements for cotton, hay, spring wheat and corn, the northward movements for winter wheat, soybeans, corn and hay, and the upward movements for hay, soybeans, spring wheat and corn.

In terms of longitude, the estimations show climate is responsible for between 7% and 50% for all crops but rice and sorghum. For rice, the estimated model predicts shifts in the opposite directions in comparison to observed estimates with over-prediction of the estimate for sorghum. For latitude, the model evaluation explains 20–36% of the northward movement in hay, corn, soybeans and winter wheat but missed the direction of movement for barley, cotton, rice, sorghum and spring wheat. For elevation, the model extrapolations shows climate is responsible for between 4% and 28% of the upward movements for all crops but rice and sorghum where it misses the direction.

### Future crop mix shift

Next we turn attention toward how crop mixes may evolve under projected climate change. Here we again use the centroids approach. For the projections, we use RCP 4.5 and RCP 8.5 for 2050 and 2090 averaged over the six climate models (CanESM2, CCSM4, CESM1-CAM5, GFDL-CM3, HadGEM2-ES, and MPI-ESM-MR)^13^.

**Figure 2:**
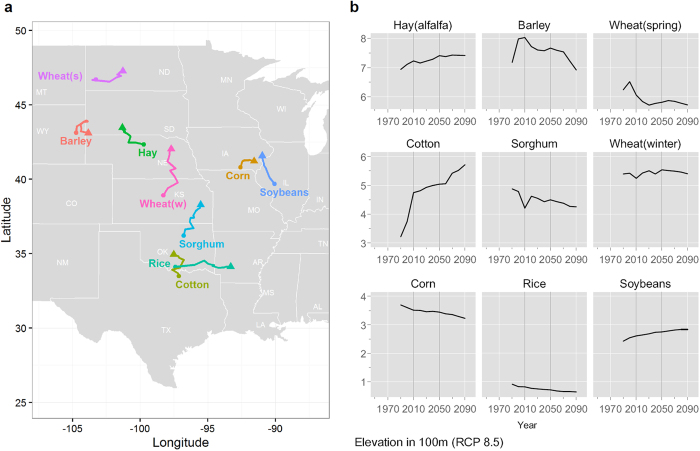
Locational shifts in production-weighted centroids and elevations for each crop under the RCP 8.5 scenario. (**a**) Solid triangles indicate the 2090 centroids for each crop under RCP 8.5 scenario. Each crop label on the plot region indicates the starting point (the 1990 centroids) for each crop, and solid lines show locus of shifts in centroids over ten-year periods. In terms of latitude changes, the production areas of hay, winter wheat, soybeans and cotton move north substantially under RCP 8.5. In terms of the changes in longitude, the production centroids of hay and cotton move west, and spring wheat, rice, and corn move east. The changes of production regions for barley and corn are expected to be less considerable. The map was generated by using maps (https://cran.r-project.org/package=maps) and ggplot2 (http://ggplot2.org) packages in R version 3.3.0 (https://www.r-project.org). (**b**) The figures show the weighted mean elevations for each crop over ten-year periods under RCP 8.5 scenario.

Table 3 presents the expected shift in centroids and elevations, and Fig. 2 shows the patterns on a US map. In terms of latitude, mixed changes are shown as alfalfa hay, barley and soybeans are expected to move to the generally drier western regions while corn, cotton, rice, sorghum, winter wheat and spring wheat move east. Under the RCP 4.5 scenario, there is not much adjustment. Cotton and rice move northward and lower in elevation (26.4% to 41.4% and −16.4% to −25.5%, respectively), while soybeans and spring wheat are likely to move north and lower moderately (7.4% to 12.4% and −7.3% to −7.9%).

Almost all of the major crops in the US in both 2020–2050 and 2060–2090 periods show the pattern of moving north (colder areas) under both RCP scenarios. Rice is the exception moving south although to a small degree (−0.5% to −0.6%). Also all crops except for rice and barley move further north under RCP 8.5 than under RCP 4.5.

## Discussion

This study examined how climate affects crop mix. To do so, we estimated how land area shares by crop are affected by climate, socioeconomic factors, and crop revenue. We find that when the annual temperature goes up, the overall proportions of cotton, rice, sorghum, and winter wheat are likely to increase with barley, corn, soybeans and spring wheat declining. We also find that increased precipitation reduces barley, sorghum, hay, and all types of wheat but increases corn, rice, and soybeans. Our results show that increasing inter-annual standard deviations of climate variables are likely to reduce the proportions for some crops: barley, rice, winter wheat and hay by larger temperature variations and corn, cotton and hay by larger precipitation variations. The results describe how farmers’ adaptation strategies for crop allocations change under altering climate.

Historically, we find that climate has contributed to shifts in production regions for several of the crops between 1970 and 2010. In terms of the future, we find that climate change as manifest in the RCP scenarios causes mix shifts for most of the major crops except corn with the movement being to the north and to higher elevations.

Although we estimated the crop mix allocations affected by various factors, there might be some omitted variables to explain the changes. We assumed that farmers are risk-neutral price takers in crop land allocations and crop yield is stable over time. Furthermore, although our results include the existing crops, we do not explain some recent crops not settled in the past. Also, we did not explicitly model agronomic managements by farmers and state-specific agricultural policies. Thus, further research would be better conducted by considering explicit policy impacts, spillover effects, and flexible model to include newly introduced crops as well as dynamic crop yields.

## Methods

To estimate shifts in crop mix, we use multinomial fractional regression (Koch^9^, Kala *et al*.^10^, and Murteira and Ramalho^11^) and estimate land shares. The estimation employs the following function:


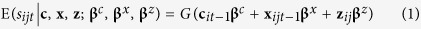


where the dependent variable *s*_*ijt*_ is the proportional land share of crop j in county i in time period t. *G*(·) is multinomial logit, **c**_*it*−1_ indicate lagged climate variables, **x**_*ijt*−1_ are net return and other socioeconomic factors, and **z**_*ij*_ are county fixed variables. The climate variables are 5-year average temperature and precipitation plus their standard deviations. The time-varying factors include five-year averages for net returns, irrigation rates, population density, and total planted area. Time-invariant factors include soil quality, average altitude of planted areas, and drought index.

To examine coefficient magnitude, we compute average partial effects (APE) that indicate the marginal impacts of an explanatory variable on crop planted acres share^14^.

Additionally, we examine the shifting pattern of crop geographic centers using a procedure like that in Reilly *et al*.^3^ and we also look at elevation. Weighting factors are production quantities evaluated with the predicted proportions multiplied by total planted acres and observed yields of each crop.

We used actual historical data and share predicted under climate change to simulate the past and future crop shifts. Details on the data used are described in the supplementary information.

## Additional Information

**How to cite this article**: Cho, S. J. and McCarl, B. A. Climate change influences on crop mix shifts in the United States. *Sci. Rep.* **7**, 40845; doi: 10.1038/srep40845 (2017).

**Publisher's note:** Springer Nature remains neutral with regard to jurisdictional claims in published maps and institutional affiliations.

## Supplementary Material

Supplementary Information

## Figures and Tables

**Table 1:** Average partial effects on proportions of planted acres.

Variables	Barley	Corn	Cotton	Rice	Sorghum	Soybeans	Wheat (winter)	Wheat (spring)	Hay (alfalfa)
Temperature (°C)	−	−	+	+	+	−	+	−	−
Precipitation (100 mm)	−	+	+	+	−	+	−	−	−
Temperature SD	−	+	+	−	.	+	−	+	−
Precipitation SD	+	−	−	.	.	+	.	+	−
Altitude (100 m)	−	+	+	.	+	−	+	−	+
Soil quality	.	.	.	.	+	+	.	.	−
PDSI	−	−	−	+	.	+	.	−	−
Irrigation rate	.	+	+	+	.	.	−	.	−
Log(Population density)	+	+	.	.	−	+	−	−	.
Log(Planted acres)	−	−	+	+	−	+	+	+	−
Net return - Barley	+	+	.	.	.	−	.	−	.
Net return - Corn	−	+	.	.	−	−	.	−	−
Net return - Cotton	.	+	+	−	−	.	−	.	.
Net return - Rice	+	+		+	.	+	+	−	.
Net return - Sorghum	+	+	−	+	+	.	−	−	−
Net return - Soybeans	−	+	−	−	.	+	−	−	.
Net return - Wheat(winter)	+	.	.	−	−	−	+	.	.
Net return - Wheat(spring)	.	−	.	−	.	+	.	+	.
Net return - Hay(alfalfa)	−	.	.	−	−	−	.	−	+

Notes: Signs of the effects with statistical significance at the 5% level are presented. Period (.) indicates the effects that are not statistically significant at the 5% level. See SI Appendix, Table S2 for details.

**Table 2:** Projected climate impacts on historical centroid shifts between 1970 and 2010.

Mean location/Crop	Centroid estimates in 1970	Centroid estimates in 2010	Shift estimates from 1970 to 2010	Projected shift from 1970 to 2010 under climate change	% of change due to climate change
	(A)	(B)	(B-A)	(D)	(D/(B-A))
***Longitude*****(+*****east***, **−*****west)***					
Hay(alfalfa)	−96.07	−101.88	−5.81	−1.78	31%
Barley	−98.27	−105.55	−7.27	−1.37	19%
Corn	−91.52	−92.50	−0.98	−0.24	25%
Cotton	−103.36	−100.53	2.82	1.41	50%
Rice	−92.70	−97.19	−4.49	2.77	−62%
Sorghum	−97.03	−96.75	0.28	0.57	204%
Soybeans	−89.07	−91.04	−1.97	−0.40	20%
Wheat(winter)	−96.59	−99.06	−2.47	−0.17	7%
Wheat(spring)	−100.52	−102.96	−2.44	−0.72	30%
***Latitude*****(+*****north***, **−*****south***)					
Hay(alfalfa)	40.80	42.21	1.42	0.29	20%
Barley	41.77	43.37	1.60	−0.27	−17%
Corn	40.71	41.21	0.50	0.12	24%
Cotton	34.46	33.87	−0.59	0.17	−30%
Rice	34.77	35.18	0.41	−0.77	−187%
Sorghum	36.05	36.44	0.39	−0.08	−21%
Soybeans	37.87	40.18	2.30	0.66	29%
Wheat(winter)	38.38	39.37	0.99	0.35	36%
Wheat(spring)	46.51	46.43	−0.08	0.16	−203%
***Elevation change in 100 m***					
Hay(alfalfa)	5.50	7.91	2.42	0.68	28%
Barley	4.89	8.56	3.67	0.13	4%
Corn	3.25	3.71	0.46	0.10	21%
Cotton	4.06	6.23	2.17	0.27	13%
Rice	0.68	0.88	0.20	−0.21	−101%
Sorghum	4.37	4.67	0.30	−0.44	−146%
Soybeans	1.85	2.68	0.82	0.20	25%
Wheat(winter)	4.63	6.13	1.51	0.17	11%
Wheat(spring)	5.32	6.66	1.35	0.31	23%

Notes: Projected shifts from 1970 to 2010 (D) indicate the differences of centroids between 1970 and 2010 only by altered climate, which were evaluated in the model with the observed climate variables (temperature, precipitation, their standard deviations and PDSI) in different periods given other variables remained at the 1970–2010 means.

**Table 3:** Predicted differences of production-weighted mean longitudes, latitudes and elevations between historical and future climate scenarios.

Mean location/Crop	Observed	Differences from 1980–2010			
	1980–2010^a^	2020–2050^b^		2060–2090^b^	
	Historical	RCP 4.5	RCP 8.5	RCP 4.5	RCP 8.5
***Longitude*** **(+*****east***, **−*****west***)					
Hay(alfalfa)	−100.31	−0.58	−0.65	−0.82	−1.00
Barley	−104.65	0.48	0.65	0.55	0.45
Corn	−92.52	0.28	0.28	0.49	0.75
Cotton	−97.44	0.46	0.52	0.66	0.17
Rice	−96.36	1.77	1.64	2.04	2.77
Sorghum	−96.54	0.35	0.41	0.62	0.91
Soybeans	−90.34	−0.42	−0.45	−0.53	−0.63
Wheat(winter)	−97.86	0.23	0.13	0.09	0.07
Wheat(spring)	−103.05	1.43	1.51	1.61	1.66
***Latitude*****(+*****north***, **−*****south***)					
Hay(alfalfa)	42.44	0.58	0.63	0.79	0.99
Barley	43.48	0.47	0.41	0.30	−0.22
Corn	40.94	0.34	0.35	0.33	0.34
Cotton	33.70	0.59	0.63	0.84	1.12
Rice	34.32	−0.17	−0.08	−0.23	−0.22
Sorghum	36.50	0.88	0.94	1.32	1.60
Soybeans	40.02	0.80	0.86	1.14	1.45
Wheat(winter)	39.33	1.36	1.48	2.03	2.50
Wheat(spring)	46.66	0.32	0.38	0.49	0.59
***Elevation in 100 m***					
Hay(alfalfa)	7.09	0.15	0.21	0.29	0.34
Barley	7.72	−0.01	−0.10	−0.19	−0.48
Corn	3.61	−0.15	−0.15	−0.22	−0.31
Cotton	3.92	1.04	1.06	1.16	1.62
Rice	0.85	−0.14	−0.13	−0.18	−0.22
Sorghum	4.66	−0.13	−0.16	−0.22	−0.35
Soybeans	2.52	0.19	0.20	0.26	0.31
Wheat(winter)	5.36	0.09	0.12	0.11	0.09
Wheat(spring)	6.29	−0.46	−0.51	−0.49	−0.49

Notes: ^a^Weighted averages based on historical observations are shown in this column.

^b^Differences of weighted averages from the historical averages are shown in these columns.
