# Supplementary material for: Climate change influences on crop mix shifts in the United States
Source: Sci Rep. 2017 Jan 18;7:40845. doi: 10.1038/srep40845 (PMC5241635; doi:10.1038/srep40845)
Supplement: Supplementary Information [file srep40845-s1.pdf]

Climate change influences on crop mix shifts in the United States

Sung Ju Cho[a,*], Bruce A. McCarl[b]

[a] Korea Rural Economic Institute, 601 Bitgaram-ro, Naju-si, Jeollanam-do, 58217, Korea

[b] Department of Agricultural Economics, Texas A&M University, College Station, TX 77843-2124, USA

* Corresponding author. Tel.: +82-61-820-2376; Fax: +82-61-820-2407; E-Mail: sungjucho@krei.re.kr.

Keywords: crop mix, locational shifts, climate change adaptation

Supplementary information

Background on estimation approach

Farmers are assumed to choose crop land shares (mix) based on maximizing expected profits. Land shares are a proportion between zero and one. We used fractional regression models [1-4] because of their ability to handle zero observations where crops are not grown in some regions.

We use the fractional multinomial logit estimation method to estimate how the climate, geophysical, and socioeconomic factors affect land allocations of multiple crops. To estimate land use shares, we used the quasi-likelihood method following Papke and Wooldridge [2] and Wooldridge [5]. In this case the specific quasi-maximum likelihood function is

$$L = \prod_{i=1}^{N} \prod_{j=1}^{J} G(\mathbf{x}_{it-1}; \boldsymbol{\beta}_j)^{s_{ijt}}$$

and the log-likelihood function of the predicted dependent variable s is

$$l_i(\boldsymbol{\beta}) = s_{i1t} \log[G(\mathbf{x}_{it-1}; \boldsymbol{\beta}_1)] + s_{i2t} \log[G(\mathbf{x}_{it-1}; \boldsymbol{\beta}_2)] + \cdots + s_{iJt} \log[G(\mathbf{x}_{it-1}; \boldsymbol{\beta}_J)] \, .$$

which is consistent since the log-likelihood function is a member of the linear exponential family (LEF) [6,7].

Note that, as indicated in Papke and Wooldridge [8], when using QMLE we need to ensure the standard errors are robust so we used heteroskedasticity-consistent robust standard errors as also discussed in Papke and Wooldridge [2].

To compare the magnitude of the coefficients, we compute average partial effects (APE) that indicate the marginal impact of a change in each of the explanatory variables on the shares of each crop in terms of total crop planted acres [9]. For continuous explanatory variables, the average partial effect of the m-th explanatory variable on the land share for crop j is expressed as

$$\frac{\partial \mathrm{E}[s_{ijt}|\mathbf{x}_i]}{\partial x_{it-1}^m} = N^{-1} \sum_{i=1}^{N} \left(\beta_j^m G_j(\mathbf{x}_{it-1}; \boldsymbol{\beta}) - G_j(\mathbf{x}_{it-1}; \boldsymbol{\beta}) \sum_{k=1}^{J-1} G_k(\mathbf{x}_{it-1}; \boldsymbol{\beta}) \beta_k^m \right)$$

where s_{ijt} is the observed land use share for crop j in county i in time t and x_{it}^m is the value of one of the continuous explanatory variables in county i in time t. For discrete explanatory variables, the average partial effect is calculated as

$$\frac{\Delta \mathrm{E}[s_{ijt}|\mathbf{x}_{it-1}]}{\Delta x_{it-1}^{m}} = N^{-1}\sum_{i=1}^{N}\left(G\left(\mathbf{x}_{it-1}^{-m}\boldsymbol{\beta}_{j}^{-m} + \boldsymbol{\beta}_{j}^{m}\right) - G\left(\mathbf{x}_{it-1}^{-m}\boldsymbol{\beta}_{j}^{-m}\right)\right)$$

where $\mathbf{x}_i^{-m}$ indicates the other explanatory variables besides x_i^m in county i.

Data and variables used

We cover nine major crops, which constitute about 96% of harvested crop lands, for 2693 counties in 41 United States and years from 1976 to 2012. The crops used are barley, corn, upland cotton, rice, sorghum, soybeans, winter wheat, spring wheat, and alfalfa hay. Alfalfa hay is considered the base crop. From the 48 contiguous US, Connecticut, Delaware, Maine, Massachusetts, New Hampshire, Rhode Island, and Vermont are excluded because the croplands are too small or there is a lack of data. The total number of observations is 99,641. Missing values are filled in with linear interpolation.

Descriptions and sources of the included variables appear in Table S1. The dependent variables are the proportional land share for each crop of county planted acres in each year and the explanatory variables consist of climate, geophysical, and socioeconomic factors.

Planted acres, harvested acres, and crop yield data were drawn from USDA NASS Quick Stats [10] from 1975 to 2012. Although hay is a perennial and would not a readily respond to current conditions, it is reasonable to assume that it responds to the 5-year average values of the explanatory variables. Wheat types vary across geographic regions and exhibit different responses to climate. Thus, we separately estimate the effects on the proportion for three types of wheat. For example, spring wheat is the most tolerant to cold weather and is used in the coldest regions while winter wheat is used in warmer areas.

Price received by farmers ($ per unit of commodity) and Yield (unit of commodity per acre) were also drawn from QuickStats but on the state level. Missing values for price are filled with the price from an adjacent location. Production cost data were drawn from USDA ERS Commodity Costs and Returns report [11]. Because classifications used in the cost and returns data differ over time and across crops, the variable costs were calculated into classes making them compatible. All the costs and prices are normalized by the Producer Prices Received Index [12] into 1990 constant dollar values.

Net returns ($ per acre) was calculated as Price received ($ per unit of commodity) multiplied by Yield (units of commodity per acre) minus Variable (Operating) cost. Counties with observations of omitted or zero total harvested acres were excluded from the estimation.

We included geophysical factors to control for the location-specific characteristics. Land capability classification [13] is used as discussed in Lubowski et al. [14]. Altitude in 100m was also included as a geographic factor.

Climate data were obtained from the United States Historical Climatology Network (USHCN) [15]. Included climate variables are annual average temperature and annual total precipitation, as well as their squared values as we assumed nonlinear trends in development of yield and profits with respect to climate variables. As shown in Mendelsohn et al. [16], both climate normals and inter-annual variations are likely to play an important role in crop mix selection. Thus, in this study, standard deviations of temperature and precipitation are also included.

Palmer drought severity index (PDSI) was drawn from National Oceanic and Atmospheric Administration (NOAA)'s National Climatic Data Center (NCDC) at the climate division level [17]. PDSI is based on the balance of moisture supply and demand and indicates the severity of a wet or dry spell with negative values indicating dry spells and positive values indicating wet spells. Since the PDSI does not consider the influence of irrigation, we also included proportion of irrigated land by county from the USDA Census of Agriculture (1969-2012) [18].

Using the quasi-maximum likelihood method, we tested several specifications regarding the temperature and precipitation terms. In the testing, we used the Akaike information criterion (AIC) and Bayesian information criterion (BIC) to compare models. In turn, we found that the model including climate squared terms is more desirable than the model without the squared terms. We also found that the inclusion of net return variables improves the model fit. On the other hand, we did not see significant improvement in including year dummies for time fixed effects.

The results of unit root tests in panel data following Breitung [19] show that the dependent and independent variables do not suffer from autocorrelation and contemporaneous correlation except for the share of soybeans and the net return of barley.

Table S1: Descriptions and Sources of Variables

Variables	Description	Source
% Barley	Land share for barley planted acres (%)	USDA NASS
% Corn	Land share for corn grain planted acres (%)	USDA NASS
% Cotton	Land share for upland cotton planted acres (%)	USDA NASS
% Rice	Land share for rice planted acres (%)	USDA NASS
% Sorghum	Land share for grain sorghum planted acres (%)	USDA NASS
% Soybeans	Land share for soybean planted acres (%)	USDA NASS
% Wheat(winter)	Land share for winter wheat planted acres (%)	USDA NASS
% Wheat(spring)	Land share for spring wheat planted acres (%)	USDA NASS
% Hay(alfalfa)	Land share for alfalfa hay harvested acres (%)	USDA NASS
Temperature	5-year average of annual mean temperature (℃)	USHCN
Precipitation	5-year average of annual total precipitation (100mm)	USHCN
Temperature SD	Standard deviation of Temperature	USHCN
Precipitation SD	Standard deviation of Precipitation	USHCN
Altitude in 100m	Altitude from the sea level (100m)	SSURGO
Soil quality	Weighted average of reverse-order land capability classifications (1 = least suitable for cultivation; …; 8 = most suitable for cultivation)	SSURGO
PDSI	Palmer drought severity index (< -4.0 = extreme drought; …; (-0.5,0.5) = normal ; …; > 4.0 = extreme wet spell)	NOAA CDO
Irrigation rate	Irrigation rate of cropland (%)	USDA NASS
Log(Population density)	Logarithm of population density (persons in an acre)	CENSTAT
Log(Planted acres)	Logarithm of total planted acres	USDA NASS
Net return – Barley	Net return of barley production per acre	Calculated
Net return – Corn	Net return of grain corn production per acre	Calculated
Net return – Cotton	Net return of upland cotton production per acre	Calculated
Net return – Rice	Net return of rice production per acre	Calculated
Net return – Sorghum	Net return of grain sorghum production per acre	Calculated
Net return – Soybeans	Net return of soybean production per acre	Calculated
Net return – Wheat(winter)	Net return of winter wheat production per acre	Calculated
Net return – Wheat(spring)	Net return of spring wheat production per acre	Calculated
Net return – Hay(alfalfa)	Revenue of alfalfa hay production per acre	Calculated

Notes: Net return for each crop ($/acre) is calculated as state price ($/unit) × county yield (unit/acre) - national cost ($/acre) for each crop.

Table S2: Statistics of variables

Variables	Mean	Std.Dev.	Min	Max
% Barley	0.03	0.08	0.00	0.97
% Corn	0.29	0.22	0.00	1.00
% Cotton	0.05	0.14	0.00	0.97
% Rice	0.01	0.06	0.00	0.97
% Sorghum	0.06	0.10	0.00	1.00
% Soybeans	0.22	0.21	0.00	0.96
% Wheat(winter)	0.18	0.21	0.00	1.00
% Wheat(spring)	0.03	0.10	0.00	0.94
% Hay(alfalfa)	0.14	0.21	0.00	1.00
Temperature	12.60	4.43	-0.71	24.24
Precipitation	9.51	3.47	0.69	29.73
Temperature SD	0.25	0.10	0.00	0.81
Precipitation SD	0.63	0.32	0.00	5.78
Altitude	4.09	4.44	0.01	28.33
Land capability class	4.75	1.35	0.00	7.25
PDSI	0.39	1.12	-4.98	6.66
Irrigation rate	0.05	0.12	0.00	1.00
Log(Population density)	4.04	1.52	-1.25	9.09
Log(Planted acres)	10.61	1.57	6.04	13.70
Net return - Barley	0.26	0.50	-0.99	5.86
Net return - Corn	1.08	1.03	-1.72	8.06
Net return - Cotton	0.13	0.92	-3.21	10.36
Net return - Rice	0.07	0.47	-1.48	14.75
Net return - Sorghum	0.38	0.51	-0.88	4.11
Net return - Soybeans	0.97	0.78	-0.74	4.24
Net return - Wheat(winter)	0.80	0.57	-0.84	6.26
Net return - Wheat(spring)	0.17	0.49	-0.56	7.59
Net return - Hay(alfalfa)	2.24	1.92	0.00	13.63

Note: Std.Dev. indicates standard deviations.

The estimates of the average partial effects on proportions of planted areas are shown in Table S3 in detail. Table 1 in the main text only shows the signs of the effects.

Table S3: Average partial effects on proportions of planted acres

Variables	Barley	Corn	Cotton	Rice	Sorghum	Soybeans	Wheat (winter)	Wheat (spring)	Hay (alfalfa)
Temperature (℃)	-0.0029***	-0.0182***	0.0183***	0.0029***	0.0103***	-0.0090***	0.0217***	-0.0061***	-0.0163***
	(0.0004)	(0.0011)	(0.0010)	(0.0005)	(0.0006)	(0.0010)	(0.0010)	(0.0005)	(0.0008)
Precipitation (100mm)	-0.0042***	0.0056***	0.0044***	0.0020***	-0.0049***	0.0163***	-0.0053***	-0.0068***	-0.0071***
	(0.0006)	(0.0017)	(0.0010)	(0.0003)	(0.0008)	(0.0016)	(0.0018)	(0.0006)	(0.0014)
Temperature SD	-0.0061**	0.0284***	0.0172***	-0.0127***	0.0044	0.0212***	-0.0386***	0.0068***	-0.0206***
	(0.0029)	(0.0084)	(0.0047)	(0.0016)	(0.0044)	(0.0060)	(0.0076)	(0.0016)	(0.0063)
Precipitation SD	0.0151***	-0.0184***	-0.0074***	0.0008	0.0057	0.0122***	-0.0066	0.0078***	-0.0100**
	(0.0024)	(0.0049)	(0.0026)	(0.0006)	(0.0041)	(0.0027)	(0.0040)	(0.0016)	(0.0049)
Altitude (100m)	-0.0012***	0.0060***	0.0134***	0.0003	0.0073***	-0.0476***	0.0185***	-0.0023***	0.0056***
	(0.0003)	(0.0020)	(0.0013)	(0.0008)	(0.0008)	(0.0023)	(0.0016)	(0.0002)	(0.0009)
Soil quality	-0.0019	-0.0007	0.0009	-0.0020*	0.0065***	0.0273***	-0.0072*	0.0014*	-0.0245***
	(0.0012)	(0.0033)	(0.0024)	(0.0011)	(0.0016)	(0.0031)	(0.0039)	(0.0008)	(0.0028)
PDSI	-0.0013***	-0.0039***	-0.0042***	0.0020***	0.0003	0.0095***	0.0006	-0.0009***	-0.0021***
	(0.0003)	(0.0009)	(0.0008)	(0.0003)	(0.0004)	(0.0009)	(0.0010)	(0.0001)	(0.0006)
Irrigation rate	0.0016	0.1095***	0.0563***	0.0400***	-0.0019	-0.0288	-0.1199***	-0.0035	-0.0560**
	(0.0083)	(0.0326)	(0.0142)	(0.0035)	(0.0116)	(0.0227)	(0.0352)	(0.0056)	(0.0222)
Log(Population density)	0.0020***	0.0112***	-0.0030*	0.0005	-0.0040***	0.0066***	-0.0105***	-0.0017***	-0.0009
	(0.0007)	(0.0024)	(0.0016)	(0.0007)	(0.0011)	(0.0022)	(0.0027)	(0.0006)	(0.0019)
Log(Planted acres)	-0.0022**	-0.0343***	0.0126***	0.0020***	-0.0100***	0.0355***	0.0256***	0.0060***	-0.0362***
	(0.0010)	(0.0029)	(0.0016)	(0.0005)	(0.0013)	(0.0023)	(0.0030)	(0.0009)	(0.0026)
Net return - Barley	0.0224***	0.0407***	-0.0078	0.0042*	-0.0003	-0.0721***	0.0107	-0.0037**	0.0059
	(0.0026)	(0.0069)	(0.0054)	(0.0025)	(0.0033)	(0.0056)	(0.0065)	(0.0018)	(0.0053)
Net return - Corn	-0.0039***	0.0774***	0.0034	0.0009	-0.0088***	-0.0550***	0.0036	-0.0048***	-0.0127***
	(0.0013)	(0.0047)	(0.0021)	(0.0008)	(0.0018)	(0.0033)	(0.0040)	(0.0009)	(0.0026)
Net return - Cotton	-0.0028*	0.0083**	0.0158***	-0.0021***	-0.0032***	0.0019	-0.0222***	-0.0023	0.0070
	(0.0017)	(0.0041)	(0.0014)	(0.0004)	(0.0010)	(0.0025)	(0.0034)	(0.0014)	(0.0049)
Net return - Rice	0.0255***	0.0373**	0.0024	0.0053***	0.0051*	0.0466***	0.0399***	-0.1689***	0.0177
	(0.0070)	(0.0173)	(0.0030)	(0.0005)	(0.0029)	(0.0076)	(0.0112)	(0.0298)	(0.0282)
Net return - Sorghum	0.0088***	0.0172***	-0.0129***	0.0071***	0.0523***	-0.0012	-0.0475***	-0.0091***	-0.0148***
	(0.0033)	(0.0055)	(0.0039)	(0.0012)	(0.0030)	(0.0046)	(0.0053)	(0.0034)	(0.0054)
Net return - Soybeans	-0.0244***	0.0377***	-0.0199***	-0.0110***	-0.0048*	0.1210***	-0.0887***	-0.0160***	0.0080*
	(0.0021)	(0.0055)	(0.0043)	(0.0012)	(0.0027)	(0.0051)	(0.0060)	(0.0022)	(0.0041)
Net return - Wheat(winter)	0.0045**	0.0085	0.0060	-0.0039**	-0.0257***	-0.0448***	0.0622***	-0.0030*	-0.0034
	(0.0022)	(0.0085)	(0.0048)	(0.0019)	(0.0038)	(0.0068)	(0.0084)	(0.0016)	(0.0049)
Net return - Wheat(spring)	-0.0036*	-0.0698***	-0.0002	-0.0506***	-0.0176	0.1197***	0.0011	0.0203***	0.0022
	(0.0022)	(0.0125)	(0.0300)	(0.0105)	(0.0146)	(0.0154)	(0.0139)	(0.0021)	(0.0063)
Net return - Hay(alfalfa)	-0.0031***	0.0002	0.0007	-0.0012**	-0.0028***	-0.0209***	-0.0005	-0.0019***	0.0300***
	(0.0007)	(0.0017)	(0.0011)	(0.0005)	(0.0007)	(0.0015)	(0.0016)	(0.0007)	(0.0014)

Notes: Standard errors via delta method are shown in parentheses and *, **, and *** indicate statistical significance at the levels 10%, 5%, and 1%, respectively.

Results 1: Effects of climate

The predicted proportions of crop planted acres over the 5-year average temperature are shown in Fig. S1. Around the 1975-2010 mean (12.5 degrees Celsius), we find that warming caused increased proportions of upland cotton, rice, sorghum, and winter wheat. On the other hand, the predicted proportions of barley, corn, soybeans, spring wheat and alfalfa hay decrease as the annual mean temperature increases from that mean. For instance, winter wheat shares start decreasing as the temperature goes beyond 15 degrees Celsius and soybean shares start decreasing beyond 12 degrees Celsius. Fig. S1 also shows the regional differences in the responses of crop allocations in regions with higher and lower temperature.

Fig. S2 contains results on the alterations in crop choice under changes in precipitation. For an average 1975-2011 mean annual precipitation (947 mm) case, more precipitation causes increasing proportions of corn, rice, and soybeans while the shares of barley, sorghum, winter wheat, spring wheat and alfalfa hay decrease.

Computing weighted centroids

The weighted averages of location variables (latitude, longitude and elevation) by using production quantity as weights can be calculated as:

$$\overline{loc}_t = \sum_{i=1}^{N} w_{it} \times loc_i = \frac{\sum_{i=1}^{N} q_{it} loc_i}{\sum_{i=1}^{N} q_{it}} \quad \text{for each } t$$

where the normalized weight is $w_{it} = q_{it}/\sum_{j=1}^{N} q_{jt}$, the production quantity is q_{it}, and the location variables set is $loc_i = \{longitude_i, latitude_i, elevation_i\}$, consisting of longitude in decimal degrees, latitude in decimal degrees and elevation in 100m in county i. We estimated the predicted production quantity of crop j in county i in time t as $\hat{q}_{it}^{j} = f_{it}^{j} G(\mathbf{x}_{it}; \hat{\boldsymbol{\beta}}) A_{it}^{j}$ where the f_{it}^{j} is the yield of crop j in county i in time t, $G_j(\mathbf{x}_{it}; \hat{\boldsymbol{\beta}})$ is the predicted probability of allocating land for crop j, and A_{it}^{j} is the total planted acre.

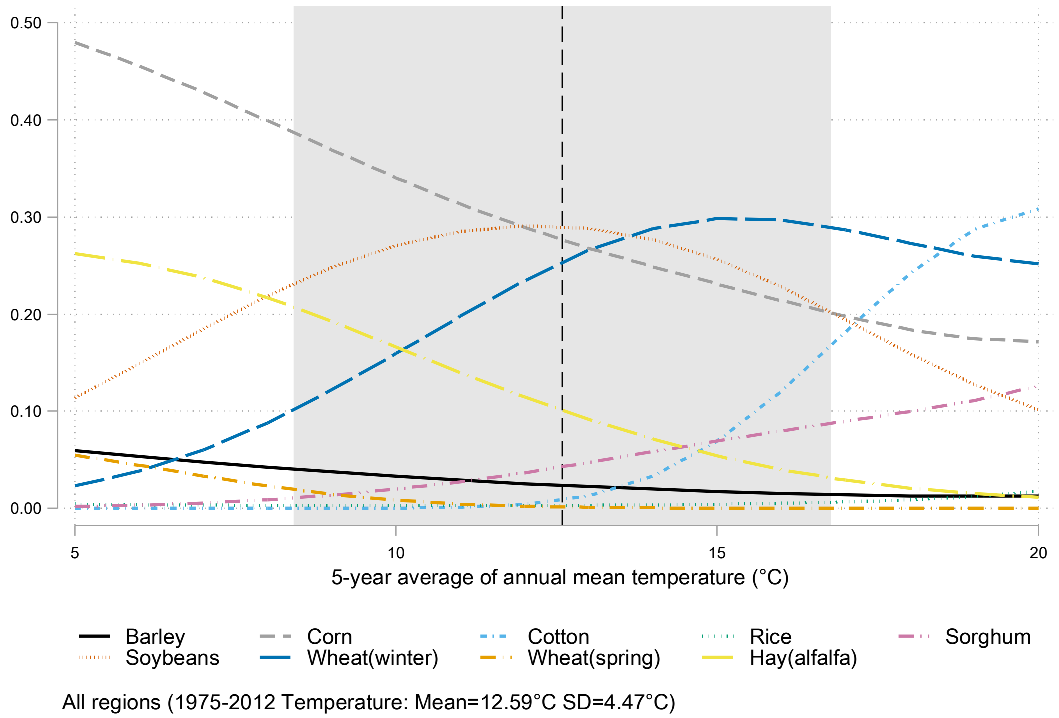

Figure S1: Predicted proportions of crop planted acres over temperature. Predicted proportions are evaluated holding other variables including precipitation at their observed values. Annual mean temperature over 1975-2012 has mean (12.6 degrees Celsius) shown as a vertical dashed line and standard deviation (4.47).

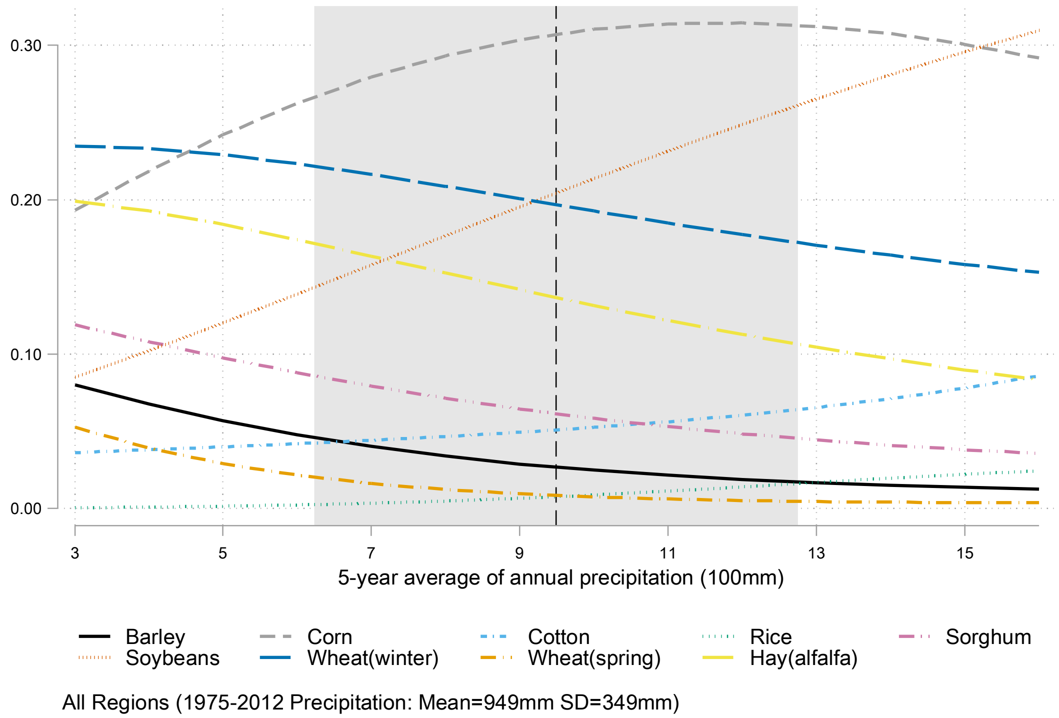

Figure S2: Predicted proportions of crop planted acres over precipitation. Predicted proportions are evaluated holding other variables including temperature at their observed values. Annual total precipitation over 1975-2012 has mean (949 mm) shown as a vertical dashed line and standard deviation (349).

Results 2: Changes in Centroids in the past

The below figure demonstrates the climate impacts on the historical cropland shifts as shown in Table 2.

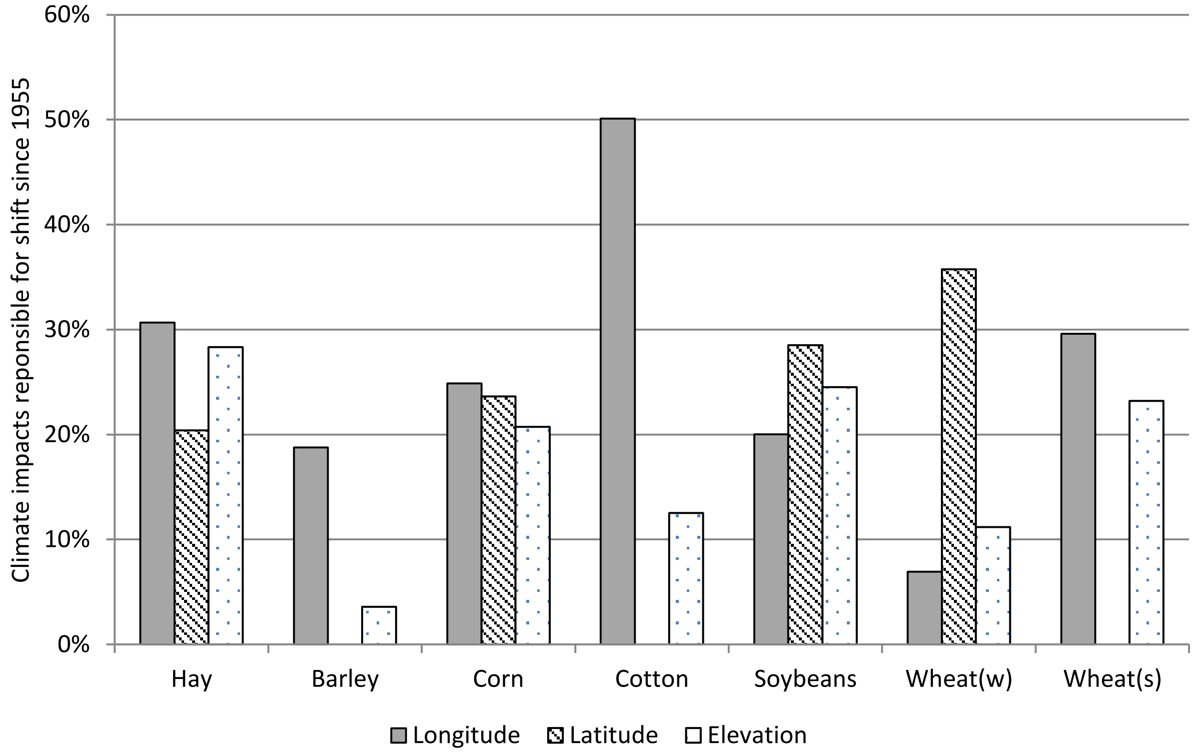

Figure S3: Projected climate impacts responsible for historical centroid shifts between 1970 and 2010. Climate is most responsible for historical longitude change of production regions for cotton, hay and spring wheat. For latitude change of production regions, winter wheat, soybeans, corn and hay are most affected by climate. In terms of elevation, climate is most responsible for change of production regions for hay, soybeans, spring wheat and corn. Negative or overpredicted values are suppressed for simplicity. See Table 2 for details.

Results 3: Centroids in the future

We obtained the projected temperature and precipitation outputs from six different climate models including CanESM2, CCSM4, CESM1-CAM5, GFDL-CM3, HadGEM2-ES, and MPI-ESM-MR from the Archive of CONUS 1/8 degree BCSD (Bias-Corrected and Spatially Downscaled) [20]. Based on the projected climate variables, we projected the weighted mean of locations variables under RCPs 4.5 and 8.5 in the 2010-2090 period.

Fig. 2 in the main text only shows trajectories of horizontal and vertical shifts. Here, the expected trails of mean elevations are presented in Fig. S4 under RCPs 4.5 and 8.5.

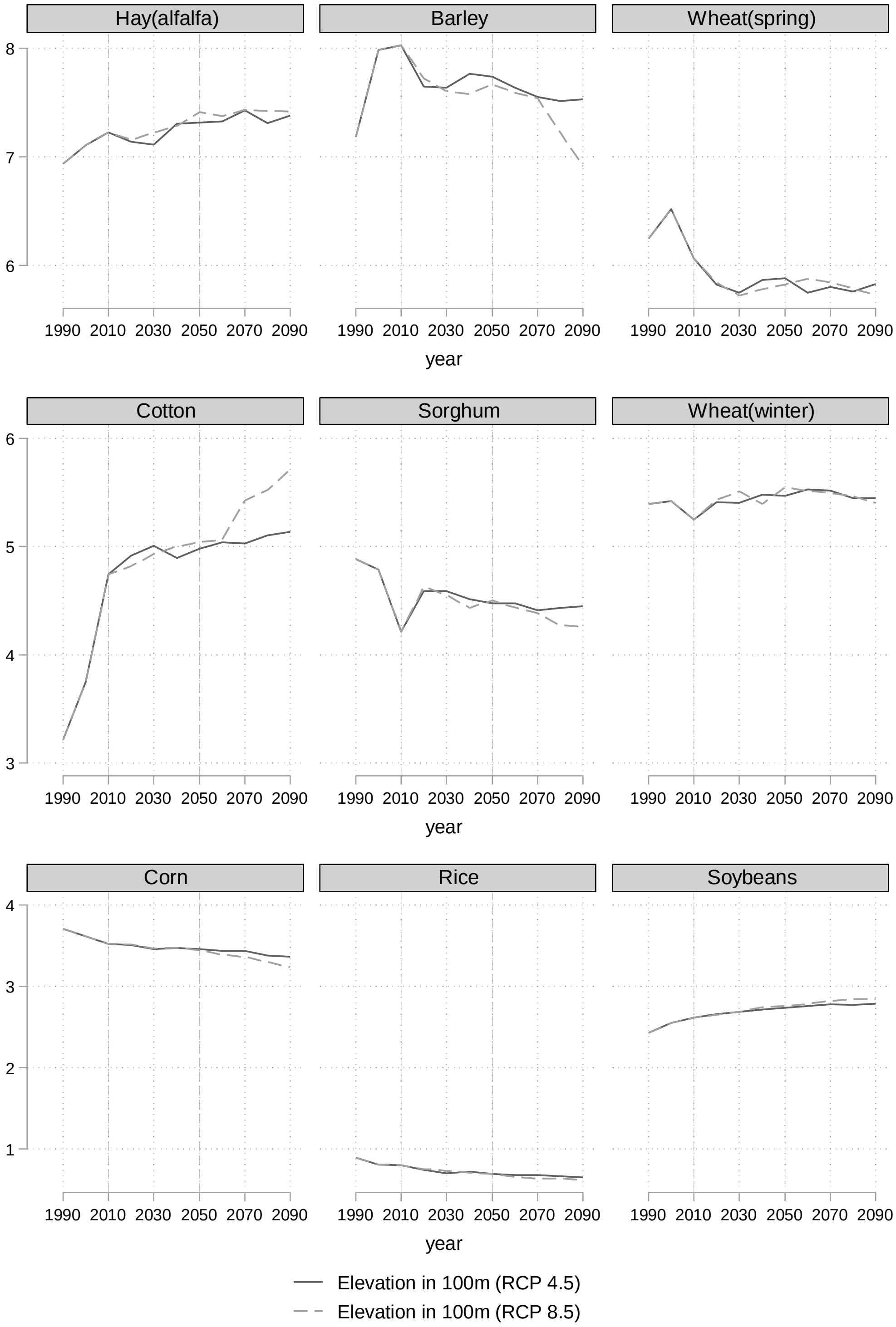

Figure S4: Change in production-weighted mean elevations for crops under RCPs 4.5 and 9.5 (10-year moving average)

References

1 Mullahy, J. & Robert, S. A. No Time to Lose: Time Constraints and Physical Activity in the Production of Health. *Review of Economics of the Household* **8**, 409-432 (2010).

2 Papke, L. E. & Wooldridge, J. M. Econometric Methods for Fractional Response Variables with an Application to 401(K) Plan Participation Rates. *J Appl Economet* **11**, 619-632 (1996).

3 Ramalho, E. A., Ramalho, J. J. S. & Murteira, J. M. R. Alternative Estimating and Testing Empirical Strategies for Fractional Regression Models. *Journal of Economic Surveys* **25**, 19-68 (2011).

4 Sivakumar, A. & Bhat, C. Fractional Split-Distribution Model for Statewide Commodity-Flow Analysis. *Transportation Research Record: Journal of the Transportation Research Board* **1790**, 80-88 (2002).

5 Wooldridge, J. M. *Econometric Analysis of Cross Section and Panel Data*. 2nd edn, (MIT Press, 2010).

6 Gourieroux, C., Monfort, A. & Trognon, A. Pseudo Maximum Likelihood Methods: Theory. *Econometrica* **52**, 681-700 (1984).

7 McCullagh, P. & Nelder, J. A. *Generalized Linear Models*. 2nd edn, (Chapman and Hall, 1989).

8 Papke, L. E. & Wooldridge, J. M. Panel Data Methods for Fractional Response Variables with an Application to Test Pass Rates. *Journal of Econometrics* **145**, 121-133 (2008).

9 Long, J. S. & Freese, J. *Regression Models for Categorical Dependent Variables Using Stata, Second Edition*. (Stata Press, 2006).

10 U.S. Department of Agriculture. *Quick Stats* https://quickstats.nass.usda.gov/ (National Agircultural Statistics Service, Washington, DC, 2013).

11 U.S. Department of Agriculture. *Commodity Costs and Returns* http://www.ers.usda.gov/data-products/commodity-costs-and-returns.aspx (Economic Research Service, 2013).

12 U.S. Department of Agriculture. *Price Indexes and Discount Rates* http://www.nrcs.usda.gov/wps/portal/nrcs/detail/national/technical/econ/prices/?cid=nrcs143_009709 (Natural Resrouces Convervation Service, 2013).

13 Klingebiel, A. A. & Montgomery, P. H. *Land-Capability Classification*. (U.S. Department of Agriculture, Soil Conservation Service, Agriculture Handbook No. 210, 1961).

14 Lubowski, R. N., Plantinga, A. J. & Stavins, R. N. Land-Use Change and Carbon Sinks: Econometric Estimation of the Carbon Sequestration Supply Function. *Journal of Environmental Economics and Management* **51**, 135-152, doi:DOI 10.1016/j.jeem.2005.08.001 (2006).

15 Menne, M. J., Durre, I., Vose, R. S., Gleason, B. E. & Houston, T. G. An Overview of the Global Historical Climatology Network-Daily Database. *Journal of Atmospheric and Oceanic Technology* **29**, 897-910, doi:10.1175/JTECH-D-11-00103.1 (2012).

16 Mendelsohn, R., Basist, A., Dinar, A., Kurukulasuriya, P. & Williams, C. What Explains Agricultural Performance: Climate Normals or Climate Variance? *Climatic Change* **81**, 85-99, doi:10.1007/s10584-006-9186-3 (2007).

17 Vose, R. S. *et al.* Improved Historical Temperature and Precipitation Time Series for U.S. Climate Divisions. *Journal of Applied Meteorology and Climatology* **53**, 1232-1251, doi:10.1175/JAMC-D-13-0248.1 (2014).

18 U.S. Department of Agriculture. *Census of Agriculture*. (National Agricultural Statistics Service, 2014).
19 Breitung, J. in *Nonstationary Panels, Panel Cointegration, and Dynamic Panels* Vol. 15 *Advances in Econometrics* 161-177 (Emerald Group Publishing Limited, 2001).
20 Brekke, L., Thrasher, B. L., Maurer, E. P. & Pruitt, T. *Downscaled CMIP3 and CMIP5 Climate and Hydrology Projections: Release of Downscaled CMIP5 Climate Projections, Comparison with Preceding Information, and Summary of User Needs* http://gdo-dcp.ucllnl.org/downscaled_cmip_projections/techmemo/downscaled_climate.pdf (2013).
